# Regional adaptation of the education in palliative and end‐of‐life Care Pediatrics (EPEC‐Pediatrics) curriculum in Eurasia

**DOI:** 10.1002/cam4.5213

**Published:** 2022-09-08

**Authors:** Michael J. McNeil, Bella Ehrlich, Taisiya Yakimkova, Huiqi Wang, Volha Mishkova, Zhanna Bezler, Ella Kumirova, Arshia Madni, Narine Movsisyan, Karen Williams, Baglan Baizakova, Marina Borisevich, Georgia Chatman, Indira Erimbetova, Ximena Garcia Quintero, Rodica Golban, Brandi Kirby, Paola Nunez, Radhikesh Ranadive, Nadezhda Sakhar, Jason Sonnenfelt, Alisa Volkova, Daniel Moreira, Stefan J. Friedrichsdorf, Joanne Wolfe, Stacy Remke, Joshua Hauser, Meenakshi Devidas, Justin N. Baker, Asya Agulnik

**Affiliations:** ^1^ St. Jude Children's Research Hospital Memphis Tennessee USA; ^2^ Warren Alpert Medical School of Brown University Providence Rhode Island USA; ^3^ Belarusian Research Center for Pediatric Oncology Hematology and Immunology Minsk Belarus; ^4^ Belarusian Clinical Center of Palliative Care for Children Minsk Belarus; ^5^ Dmitry Rogachev National Research Center of Pediatric Hematology Oncology and Immunology Moscow Russia; ^6^ Russian Scientific Center of Roengenology and Radiology Moscow Russia; ^7^ Pyrogov Medical University Moscow Russia; ^8^ Morozovskaya Children's City Clinical Hospital Moscow Russia; ^9^ N.N. Blokhin National Medical Research Center of Oncology Moscow Russia; ^10^ Yerevan State Medical University After Mkhitar Heratsi Yerevan Armenia; ^11^ George Washington University The Milken Institute School of Public Health Washington, District of Columbia USA; ^12^ The Republican Center for Hematology and Blood Transfusion Tashkent Uzbekistan; ^13^ Institute of Oncology of Republic of Moldova Moldova Chisinau; ^14^ Republican Scientific and Practical Center for Pediatric Surgery Minsk Belarus; ^15^ Raisa Gorbacheva Memorial Research Institute for Pediatric Oncology Hematology and Transplantation St. Petersburg Russia; ^16^ Benioff Children's Hospitals University of California San Francisco San Francisco California USA; ^17^ Dana Farber Cancer Institute Boston Massachusetts USA; ^18^ University of Minnesota Minneapolis Minnesota USA; ^19^ Northwestern University Chicago Illinois USA

**Keywords:** education, Eurasia, pediatric oncology, pediatric palliative care

## Abstract

**Background:**

Pediatric palliative care (PPC) is a priority to improve pediatric hematology oncology (PHO) care in Eurasia. However, there are limited regional opportunities for PPC education. We describe the adaptation and implementation of a bilingual end‐user Education in Palliative and End‐of‐Life Care (EPEC)‐Pediatrics course for PHO clinicians in Eurasia.

**Methods:**

Due to COVID‐19, this course was delivered virtually, consisting of prerecorded, asynchronous lectures, and a bilingual workshop with interactive lectures and small group sessions. A pre–postcourse design was used to evaluate the knowledge acquisition of the participants including their knowledge alignment with World Health Organization (WHO) guidance, ideal timing of palliative care, and comfort in providing palliative care to their patients. Questions were mostly quantitative with multiple choice or Likert scale options, supplemented by free‐text responses.

**Results:**

A total of 44 (76%) participants from 14 countries completed all components of the course including pre‐ and postcourse assessments. Participant alignment with WHO guidance improved from 75% in the pre‐ to 90% in the postcourse assessments (*p* < 0.001). After participation, 93% felt more confident controlling the suffering of children at the end of life, 91% felt more confident in prescribing opioids and managing pain, and 98% better understood how to hold difficult conversations with patients and families. Most participants (98%) stated that they will change their clinical practice based on the skills and knowledge gained in this course.

**Conclusions:**

We present a successful regional adaptation of the EPEC‐Pediatrics curriculum, including novel delivery of course content via a virtual bilingual format. This course resulted in significant improvement in participant attitudes and knowledge of PPC along with an understanding of the ideal timing of palliative care consultation and comfort in providing PPC to children with cancer. We plan to incorporate participant feedback to improve the course and repeat it annually to improve access to high‐quality palliative care education for PHO clinicians in Eurasia.

## Graphical abstract

A lack of formal education in pediatric palliative care for clinicians caring for children in resource‐constrained settings remains a significant challenge. We successfully adapted the Education in Palliative and End‐of‐Life Care (EPEC) ‐ Pediatrics course for clinicians in Eurasia and demonstrate the course significantly improved provider knowledge and comfort in palliative care principles.

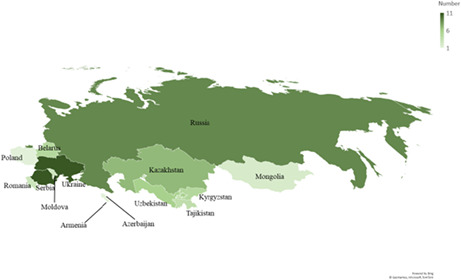

## 1 INTRODUCTION

The World Health Organization (WHO) defines palliative care as the prevention and relief of physical, developmental, psychosocial, and spiritual suffering of patients and their families facing life‐threatening illness.^1^ Early integration of palliative care is recognized by the WHO as an “ethical responsibility” for children with life‐threatening illness regardless of resource constraints.^2^, ^3^ Worldwide, more than 21 million children would benefit from pediatric palliative care (PPC) services,^4^ however, access to PPC is limited, especially in low‐ and middle‐income countries (LMICs).^5^, ^6^, ^7^ Children with cancer are a unique patient population who experience high symptom burden resulting in poor quality of life.^8^, ^9^, ^10^, ^11^, ^12^ Early integration of PPC into childhood cancer care has been shown to improve symptom management, quality of life, communication, and reduce patient and caregiver suffering.^13^, ^14^, ^15^

While there are structural barriers to PPC delivery in resource‐constrained settings, lack of formal education in PPC remains a significant challenge.^16^, ^17^, ^18^ A potential intervention is the Education in Palliative and End‐of‐life Care (EPEC)‐Pediatrics curriculum, a 24‐module course addressing principles of PPC including pain and symptom management, grief and bereavement, and communication, delivered via a combined online‐ and in‐person training. In addition to PPC content, adult learning theory is included for participants to become “Trainers” and disseminate course content to colleagues.^19^, ^20^, ^21^, ^22^, ^23^, ^24^ Since 2012, 1517 clinicians from 100 countries/territories from all six continents have participated in this curriculum, and 1027 became EPEC‐Pediatric “Trainers.” The curriculum has been adapted for large‐scale dissemination in Canada and Latin America with translation to French and Spanish.^19^, ^20^, ^21^, ^22^ In addition to “Train‐the‐Trainer” courses, EPEC‐Pediatrics also offers “End‐User” courses which teach principles of PPC to a multidisciplinary audience to improve direct clinical care.

The Eurasian Regional Program, or EurADO (Eurasian Alliance in Pediatric Oncology), is a collaboration between St. Jude Children's Research Hospital and pediatric hematology‐oncology centers, foundations, and clinicians in 15 countries in Central Asia and Eastern Europe to improve care for children with cancer in Eurasia.^25^ In 2017, EurADO identified palliative care as a regional priority.^26^, ^27^ To study barriers to palliative care integration, EurADO conducted the “Assessing Doctors' Attitudes on Palliative Treatment” (ADAPT) study in 11 Eurasian countries. Identified barriers included delayed integration of PPC into pediatric oncology practice, low access to home‐based PPC services, and misalignment of physician knowledge with WHO guidance.^28^, ^29^ This misalignment included considering palliative care as synonymous with end‐of‐life care, concerns that involvement of PPC in patient care increased parental anxiety, and lack of comfort addressing physical and emotional symptoms of children with cancer. Subsequently, a virtual EPEC‐Pediatrics end‐user course was developed to teach PPC principles to Eurasian clinicians emphasizing gaps identified by the ADAPT study supplemented with a participant needs assessment.

The objective of this report is to describe the impact of the adapted EPEC‐Pediatrics course on participant knowledge of PPC including alignment with WHO guidance, understanding of standard components of palliative care, ideal versus actual timing of palliative care, and comfort level in providing palliative care in the clinical setting. Additionally, we assessed the effectiveness and feasibility of a novel bilingual virtual course format.

## 2 METHODS

### 2.1 IRB approval

This study was approved by the Institutional Review Board at St. Jude Children's Research Hospital as category 2 exempted research including educational tests and survey procedures [SJ‐EPECPEDS (FWA00004775)]. Participation in the course resulted in implied consent.

### 2.2 Course design

The EPEC‐Pediatrics Eurasia course was developed based on findings from the Eurasian ADAPT study supplemented with input from the EurADO supportive care working group including regional experts in pediatric oncology, hospice and palliative medicine, pain medicine, and medical education.^28^, ^29^ The specific course learning objectives were to (1) understand the elements of palliative care and recognize its importance in childhood cancer care, (2) become proficient in practical applications of pain and symptom management, (3) gain skills in effective communication with patients and families, and (4) identify tools for self‐care. The EurADO working group evaluated ADAPT data and EPEC‐Pediatrics content to identify 10 modules as high‐yield topics for the region (Table S1). Additionally, these 10 modules were adjusted to account for the unique cultural context of the region by regional palliative care experts. For example, some medications were added or omitted based on availability in the region, or sensitivity to cultural views of discussion of prognosis with the child was considered in developing the material.

### 2.3 Course delivery

The 10 EPEC‐Pediatrics modules were recorded as didactic lectures by three palliative care experts from St. Jude (MM, JB, and KW); slides and voice‐over audio were then translated into Russian (TY and VM). The course was composed of 3 weeks of online content (15 total hours, March 1–March 19, 2021) via Cure4Kids, a free web‐based distance‐learning platform with an integrated learning management system.^30^ A subsequent 5‐day synchronous virtual workshop (10 total hours, March 22–26, 2021) using the videoconferencing platform Zoom (See Table S2).^31^ Each workshop day was composed of 1 h of interactive didactic lecture led by a palliative care expert with simultaneous translation followed by the second hour of small group practical sessions of skill‐development activities led by EPEC‐Pediatrics‐trained Eurasian facilitators in Russian or English. An English‐speaking EPEC‐Pediatrics expert co‐facilitator assisted the small group with simultaneous translation to allow for bidirectional discussion. Examples of the skill‐development activities included a case‐based experience teaching the principles of dyspnea, role play for communication, and wellness exercises during the self‐care small group sessions.

### 2.4 Course participants

Course participants were physicians, nurses, and psychologists who care for children with cancer recruited from Eurasian institutions that collaborate with the St. Jude Global Eurasian Regional Program^25^ or the World Health Organization Global Initiative in Childhood Cancer (WHO GICC).^32^ Letters of invitation were sent to collaborating regional leaders who distributed the invitation for participation in the course to their colleagues.

### 2.5 Survey development

We used a pre–postassessment design to evaluate the participant experience .^33^ Assessment items were developed based on the ADAPT survey.^28^, ^29^ The precourse assessment included demographics and questions assessing alignment with WHO guidance^1.^through 15 statements that agreed or disagreed with WHO guidelines.^28^ Additional sections assessed understanding of standard accepted components of palliative care, ideal versus actual timing of palliative care consultation, and comfort in providing palliative care to their patients (see Figure S1). The assessment was developed in English, translated to Russian, reviewed by regional experts, and back‐translated to ensure construct consistency.^34^ The postcourse assessment included similar questions to the precourse assessment with the addition of questions evaluating participant confidence in delivering components of palliative care after the course and an overall course evaluation (see Figure S2). Both assessments were mostly quantitative with multiple‐choice or Likert‐scale options, supplemented by several free‐text questions (See Table S3 for Course Conceptual Framework). The assessments were distributed using the Qualtrics electronic platform,^35^ with the precourse assessment in February 2021 and the postcourse assessment in April 2021.

### 2.6 Statistical analysis

Descriptive statistics were used to summarize participant demographics. Differences in pre‐ and postassessment samples were identified using McNemar's test and Symmetry test. As in the ADAPT study, the 5‐point Likert scale was collapsed into two categories (disagree and agree) to compare the pre‐ and postresults of the 15 statements assessing alignment to WHO guidance, with the neutral response categorized as not in alignment.^28^ Each participant's percent alignment to WHO guidance was then calculated as the number of statements in alignment out of 15 total, and the mean percent alignment was calculated for the pre‐ and postcourse assessments. McNemar's test was used for multiple‐choice questions assessing the timing of palliative care consultation. The symmetry test was used to compare comfort levels in providing palliative care pre‐ and posttest; delta change was calculated by the difference between posttest and pretest means. SAS software, version 9.4 was used for all quantitative analyses^36^ and a *p‐*value of <0.05 was considered statistically significant. The free‐text responses were translated to English from Russian by a bilingual native speaker (BE). Due to the concise nature of free‐text responses, thematic content analysis was conducted by one author (MJM). Responses were organized according to the presence of specific concepts that were subsequently grouped into themes for content analysis.

## 3 RESULTS

### 3.1 Participant demographics

**FIGURE 1:**
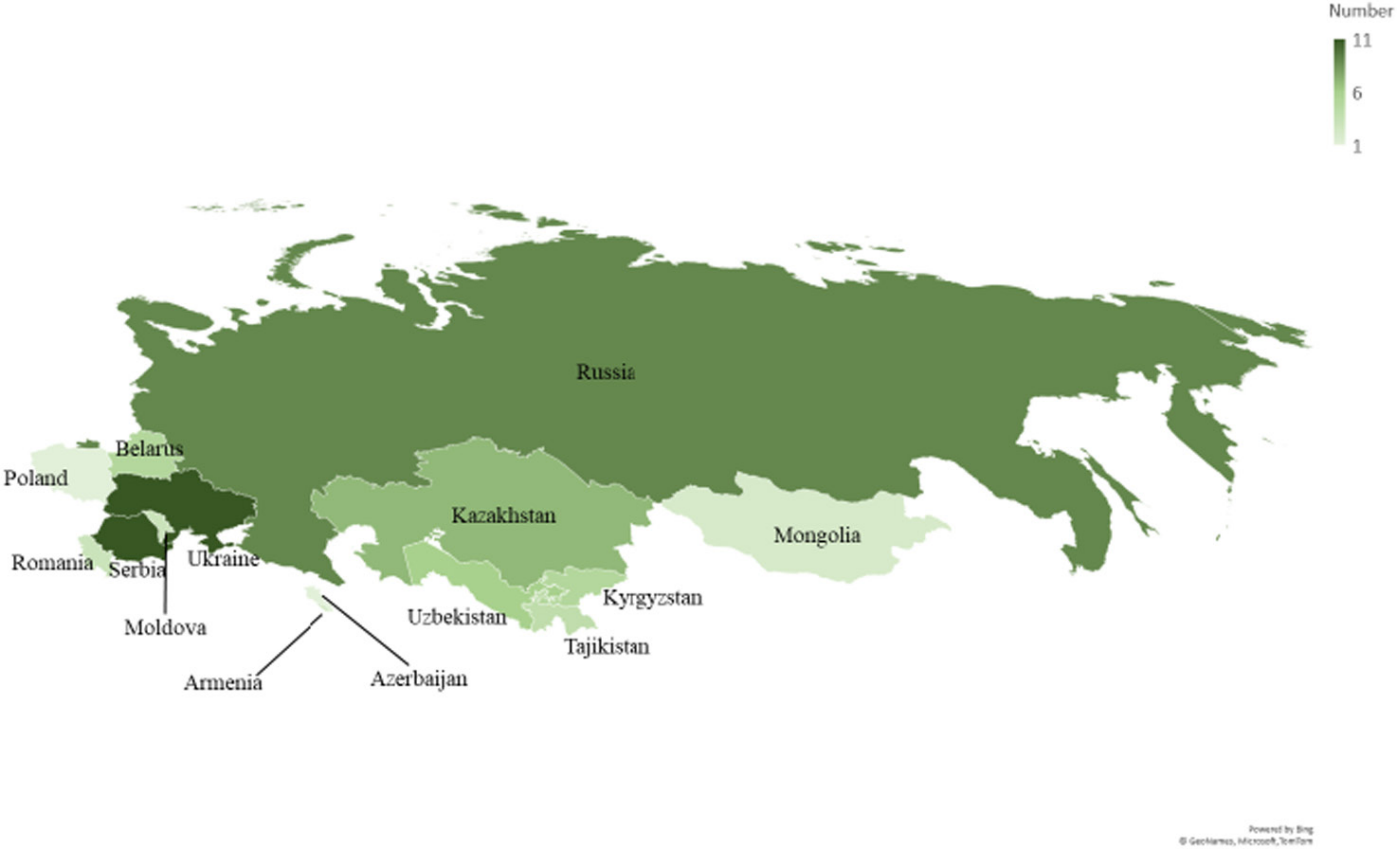
Participating countries from Eurasia: Participating countries with a darker color associated with a larger number of participants per country.

Seventy clinicians from 23 institutions in 14 countries applied to the course, with 58 ultimately enrolling and 47 submitting all course components (see Table S4 for demographics of participants who did not complete the course). Three participants had incomplete pre‐ or postcourse assessments so 44 (75.9%) participants were used for final analysis (Figure 1). Most were pediatric hematologists/oncologists (63.6%); few reported previous palliative care training (15.9%) and most did not have access to PPC consultation at their institution (63.6%). Most participants (86.4%) had at least one patient die, with over a third (36.4%) having more than five patients die in their care within the previous year (Table 1). Almost half (45.4%) of participants felt burdened by their inability to control the suffering of children at the end of life, 20.5% felt burned out by their work, and 15.9% felt more callous toward people since starting their current work (Table 1).

**TABLE 1:** Participant demographics

Demographics overall sample	(*n* = 44), No. (%)
Country	
Armenia	1 (2.3)
Azerbaijan	1 (2.3)
Belarus	5 (11.4)
Kazakhstan	5 (11.4)
Moldova	2 (4.5)
Poland	1 (2.3)
Romania	6 (13.6)
Russia	8 (18.2)
Serbia	2 (4.5)
Tajikistan	2 (4.5)
Ukraine	6 (13.6)
Uzbekistan	5 (11.4)
Age	
<35	17 (38.6)
35–50	21 (47.7)
51–65	6 (13.6)
>65	0
Sex	
Female	36 (81.8)
Male	8 (18.2)
Primary medical specialty	
Pediatric hematology and/or oncology	28 (63.6)
Pediatric anesthesia/ intensive care	5 (11.4)
Pediatric palliative care	3 (6.8)
General pediatrician	1 (2.3)
Other: (please specify)^a^	7 (15.9)
Primary institution	
Children's Hospital	23 (52.3)
Cancer Hospital	19 (43.2)
Children's Hospice	1 (2.3)
Other: (please describe)^b^	1 (2.3)
Years of experience	
0–10 years	18 (40.9)
≥11 years	26 (59.1)
Previous training in palliative care	
No	37 (84.1)
Yes	7 (15.9)
Access to palliative care consultation	
No	28 (63.6)
Yes	16 (36.4)
I have felt burdened by my inability to control the suffering of children at the end of life	
Disagree	5 (11.4)
Sometimes	19 (43.2)
Agree	20 (45.4)
I feel burned out by my work	
Disagree	12 (27.3)
Sometimes	23 (53.3)
Agree	9 (20.5)
I have become more callous toward people since I took this job	
Disagree	25 (56.8)
Sometimes	12 (27.3)
Agree	7 (15.9)
How many pediatric patients in your care (<18 years old) died in the last 12 months?	
0 patients	6 (13.6)
1–5 patients	22 (50.0)
≥6 patients	16 (36.4)

Demographics overall sample	(*n* = 44), No. (%)
Country	
Armenia	1 (2.3)
Azerbaijan	1 (2.3)
Belarus	5 (11.4)
Kazakhstan	5 (11.4)
Moldova	2 (4.5)
Poland	1 (2.3)
Romania	6 (13.6)
Russia	8 (18.2)
Serbia	2 (4.5)
Tajikistan	2 (4.5)
Ukraine	6 (13.6)
Uzbekistan	5 (11.4)
Age	
<35	17 (38.6)
35–50	21 (47.7)
51–65	6 (13.6)
>65	0
Sex	
Female	36 (81.8)
Male	8 (18.2)
Primary medical specialty	
Pediatric hematology and/or oncology	28 (63.6)
Pediatric anesthesia/ intensive care	5 (11.4)
Pediatric palliative care	3 (6.8)
General pediatrician	1 (2.3)
Other (please specify)^a^	7 (15.9)
Primary institution	
Children's Hospital	23 (52.3)
Cancer Hospital	19 (43.2)
Children's Hospice	1 (2.3)
Other (please describe)^b^	1 (2.3)
Years of experience	
0–10 years	18 (40.9)
≥11 years	26 (59.1)
Previous training in palliative care	
No	37 (84.1)
Yes	7 (15.9)
Access to palliative care consultation	
No	28 (63.6)
Yes	16 (36.4)
I have felt burdened by my inability to control the suffering of children at the end of life	
Disagree	5 (11.4)
Sometimes	19 (43.2)
Agree	20 (45.4)
I feel burned out by my work	
Disagree	12 (27.3)
Sometimes	23 (53.3)
Agree	9 (20.5)
I have become more callous toward people since I took this job	
Disagree	25 (56.8)
Sometimes	12 (27.3)
Agree	7 (15.9)
How many pediatric patients in your care (less than 18 years old) died in the last 12 months?	
0 patients	6 (13.6)
1–5 patients	22 (50.0)
≥6 patients	16 (36.4)

a Other specialties: Nurses, psychologists.

b Other institution: General Hospital.

### 3.2 Alignment with WHO

The mean participant alignment with WHO guidance prior to the workshop was 74.5% (range 25.0%–97.7%, standard deviation 22.1%) which improved postcourse to a mean alignment of 90.3% (range 52.3%–100.0%, standard deviation [SD] of 12.1%, *p* < 0.01). Of the 15 WHO statements, 8 had a statistically significant improvement between the pre‐ and postcourse assessments; the remaining 7 had precourse WHO alignment of over 89.6% (Table 2). The individual median improvement for alignment was 15.9 percentage points (range: 2.2–40.9%). The four most common precourse misconceptions were (1) it is difficult to know when a patient with cancer would most benefit from meeting the palliative care team, (2) palliative care is synonymous with “end‐of‐life” care, (3) palliative care for children with cancer can be delivered by healthcare workers of all disciplines, not only by palliative care specialists and (4) early consultation with palliative care causes increased parental burden and anxiety. Participant scores improved by at least a 20‐percentage points in the postcourse evaluation.

**TABLE 2:** Percent change in alignment with WHO guidance before and after EPEC pediatrics course

Statements	Pre (*N* = 44)	Post (*N* = 44)	Delta between Post and Pre	*p* value
	It is difficult to know when a patient with cancer would most benefit from meeting the palliative care team (collapsed neutral into agree)			
Disagree^a^	17 (38.6)	35 (79.5)	40.9	<0.001*
Agree	27 (61.4)	9 (20.5)		
	Palliative care is synonymous with “end‐of‐life” care (collapsed neutral into agree)			
Disagree^a^	23 (52.3)	37 (84.1)	31.8	<0.001*
Agree	21 (47.7)	7 (15.9)		
	Palliative care for children with cancer can be delivered by healthcare workers of all disciplines, not only by palliative care specialists (collapsed neutral into disagree)			
Disagree	19 (43.2)	6 (13.6)		0.002*
Agree^a^	25 (56.8)	38 (86.4)	29.6	
	Early consultation with palliative care causes increased parental burden and anxiety (collapsed neutral into agree)			
Disagree^a^	11 (25.0)	23 (52.3)	27.3	0.005*
Agree	33 (75.0)	21 (47.7)		
	Palliative care is incompatible with curative care (collapsed neutral into agree)			
Disagree^a^	32 (72.7)	43 (97.7)	25	0.002*
Agree	12 (27.3)	1 (2.3)		
	Involving palliative care suggests the oncologist has failed in the mission to cure the patient (collapsed neutral into agree)			
Disagree^a^	34 (77.3)	42 (95.5)	18.2	0.01*
Agree	10 (22.7)	2 (4.5)		
	Involving the palliative care team early has negative effects on the relationship between the oncologist and the patient and family (collapsed neutral into agree)			
Disagree^a^	34 (77.3)	41 (93.2)	15.9	0.03*
Agree	10 (22.7)	3 (6.8)		
	Palliative care is appropriate at any stage of treatment in a child with high‐risk cancer (collapsed neutral into disagree)			
Disagree	12 (27.3)	5 (11.4)		0.07*
Agree^a^	32 (72.7)	39 (88.6)	15.9	
	Palliative care can be integrated with disease‐directed therapy (collapsed neutral into disagree)			
Disagree	6 (13.6)	2 (4.5)		0.15*
Agree^a^	38 (86.4)	42 (95.5)	9.1	
	Involvement of palliative care undermines the role of the pediatric oncologist as the physician in charge of patient care (collapsed neutral into agree)			
Disagree^a^	40 (90.9)	44 (100.0)	9.1	0.045*
Agree	4 (9.1)	0		
	Early integration of palliative care for all children diagnosed with cancer would decrease patient suffering (collapsed neutral into disagree)			
Disagree	3 (6.8)	0		0.08*
Agree^a^	41 (93.2)	44 (100.0)	6.8	
	Early integration of pediatric palliative care with cancer care would improve interdisciplinary communication (collapsed neutral into disagree)			
Disagree	4 (9.1)	2 (4.5)		0.41*
Agree^a^	40 (90.9)	42 (95.5)	4.6	
	Children with cancer who receive palliative care die earlier than those who do not (collapsed neutral into agree)			
Disagree^a^	41 (93.2)	43 (97.7)	4.5	0.32*
Agree	3 (6.8)	1 (2.3)		
	Children with advanced and incurable cancer often suffer at the end of life (collapsed neutral into disagree)			
Disagree	3 (6.8)	3 (6.8)		1.00*
Agree^a^	41 (93.2)	41 (93.2)	0	
	Involvement of palliative care during cancer therapy gives greater attention to the quality ofss life and symptom management (e.g., pain, constipation, dyspnea, fatigue) (collapsed neutral into disagree)			
Disagree	1 (2.3)	2 (4.5)		0.56*
Agree^a^	43 (97.7)	42 (95.5)	−2.2	

^a^ Correct response. Collapsed into two categories (collapse neutral into incorrect response based on whether agree or disagree is the correct answer or not): Statements organized from the largest positive delta change toward alignment to WHO guidance answer to the lowest delta change toward WHO guidance.

* McNemar's Test.

### 3.3 Components and timing of palliative care

**FIGURE 2:**
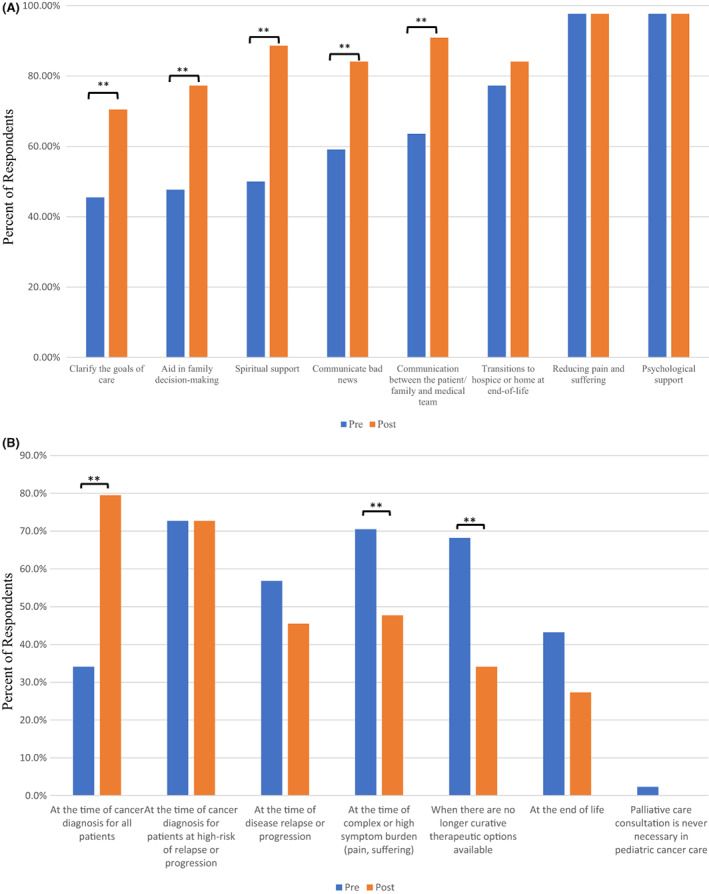
(A) Changes in Participation Perception of Palliative Care Role After EPEC Pediatrics Course: Results of pre‐ and postcourse assessments of participant descriptions of the role of palliative care for children with cancer. The precourse survey results are in blue and the postcourse survey results are in orange. Statistically significant changes in responses occurred for those roles discussing goals of care, communication, and decision‐making among others (*p* < 0.05). (B) Changes in Ideal Timing of Palliative Care After EPEC Pediatrics Course: Ideal timing of initial palliative care consultation for participants in both the precourse survey and postcourse survey responses. Respondents were instructed to choose all options that applied. The figure shows the percentage of physicians who indicated each option and the statistically significant difference between the two groups. Comparing the earliest selected timing option (converted to a numeric scale), physicians indicated that the ideal timing for an initial palliative care consultation for a child.

Participants also broadened their description of the components of palliative care for children with cancer pre‐ and postcourse (Figure 2A). Approximately 50% or less of respondents identified certain components as a part of palliative care in the precourse assessments, which were significantly higher after the course, including spiritual support to the patient and family (50.0% vs. 88.6%, *p* < 0.01), aid in family decision‐making around treatment options (47.7% vs. 77.3%, *p* < 0.01), and help clarify goals of care for the patient and family (45.5% vs. 70.5%, *p* < 0.01).

When asked about the ideal timing of palliative care consultation for a child with cancer assuming unlimited resources, more participants felt palliative care should be involved from diagnosis (79.5%) in the postcourse compared with the precourse assessment (34.1%, *p* < 0.01, Figure 2B). Additionally, in the postcourse assessment fewer participants stated that the ideal timing for consultation was at the time of complex or high symptoms compared with the precourse assessment (70.5% vs. 47.7%, *p* < 0.01).

When asked to provide examples of something they will do differently in the future, many participants emphasized the importance of implementing palliative care earlier in the patient's care: “For me, the statement that the involvement of a palliative team is possible at the time of diagnosis was a discovery. I agree that talking with parents about palliative care early will facilitate communication at different stages of the disease. I am going to inform doctors about this, to spread the necessary information about palliative for parents. It's not easy, because the mentality and attitude towards the very word ‘palliative’ is not very simple in our country, and the ‘doctor‐patient’ communication is very upsetting” (Ukraine) (Table S5).

### 3.4 Comfort and confidence in providing palliative care to patients

In the precourse assessment, 59.1% of participants reported feeling comfortable assessing and treating the physical needs of pediatric patients with serious incurable illness, which increased to 75.0% after the course (*p =* 0.03) (Table S6). While not statistically significant, there were improvements in the postcourse comfort in addressing the emotional needs of patients (72.7% pretest, 86.4% posttest, *p =* 0.2) and in addressing the grief and bereavement of families (43.2% pretest, 68.2% posttest, *p =* 0.7).

After participating in the course, most participants felt more confident in their ability to control the suffering of children at the end‐of‐life (93.2%) and to prescribe opioids and manage pain (90.9%); they also better understood possible solutions to providing palliative care for children with cancer (97.7%) and how to hold difficult conversations with patients and families (97.7%) (Table 3). In free‐text responses, many participants described the importance of self‐care and the prevention of burnout as key takeaways from the course; “I have been working in oncology for 28 years. I keep in touch with many already cured patients, as well as with the parents of children who have died. I value them, but I understand that I need to learn to love myself and devote more time to my family.” (Belarus) (Table S5).

**TABLE 3:** Percent confidence in patient care after participation in EPEC pediatrics course

Postcourse survey	
I feel more confident in my knowledge of opioid prescription and pain management for children with cancer	
Disagree	1 (2.3)
Neutral	3 (6.8)
Agree	40 (90.9)
I feel more confident in my ability to control the suffering of children at the end of life	
Disagree	0
Neutral	3 (6.8)
Agree	41 (93.2)
I better understand possible solutions on how to provide better palliative care for children with cancer	
Disagree	0
Neutral	1 (2.3)
Agree	43 (97.7)
I better understand how to hold difficult conversations with patients and their families and participate in decision‐making and planning for the end of life	
Disagree	0
Neutral	1 (2.3)
Agree	43 (97.7)
The course provided me with new knowledge and skills that are relevant to my clinical practice	
Disagree	0
Neutral	0
Agree	44 (100.0)
The course provided me with knowledge and skills that will CHANGE my clinical practice	
Disagree	0
Neutral	1 (2.3)
Agree	43 (97.7)
In terms of your expectations, the course was	
Below expectations	2 (4.5)
Met expectations	16 (36.4)
Above expectations	26 (59.1)

### 3.5 Course assessment

All participants reported the course provided them with new skills relevant to their clinical practice, and 97.7% stated that they will change their clinical practice based on knowledge gained in the course. Finally, 95.5% of participants stated that the course met or exceeded expectations (Table 3).

In free‐text responses, several participants described plans to apply the pain and symptom management, psychosocial support, and communication skills gained during the course (Table S5). When asked what they enjoyed most about the course, some participants described specific lectures or small group skill‐development sessions. The most common theme, however, focused on the unique structure of the course and the benefit of the small‐group skill‐development activities where participants were able to learn from content experts and from each other: “I liked the possibility of hearing all the voices, with less or more experience, and the fact that there was no bad answer” (Romania).

Suggestions to improve the course included using more case examples, increasing time for discussion, and allowing more interaction with other participants. Other participants were hopeful that this course could be held in‐person in the future.

## 4 DISCUSSION

The benefits of early integration of palliative care in the management of children with life‐threatening conditions have been widely described, especially in children with cancer.^9^, ^10^, ^11^, ^12^, ^37^ However, significant barriers to early integration of PPC exist, especially in LMICs.^16^, ^18^, ^38^, ^39^ The current paper describes a successful approach to address some of these barriers through the implementation of a novel bilingual virtual end‐user course. Following this course, participants demonstrated improvement in understanding of ideal timing of palliative care integration, alignment with WHO guidance, use of multimodal analgesia, and confidence in communication and self‐care.

*Earlier studies by our research team* demonstrated physicians with previous training in PPC felt more comfortable addressing the palliative care needs of their patients and reported feeling less burdened by their work.^28^, ^29^ Similarly, course participants nearly unanimously reported improvement in their self‐perceived ability to control the suffering of children under their care, provide integrated palliative care, and conduct difficult conversations. These findings have important potential implications for addressing the highly prevalent and urgent issue of burnout and compassion fatigue among clinicians caring for children with cancer – especially during the COVID19 pandemic as healthcare professionals are strained by increasing workload and uncertainty from the ongoing pandemic.^40^, ^41^, ^42^ Our successful experience may be used to guide future regional adaptation of the EPEC‐Pediatrics curriculum to improve clinician knowledge and engage collaboration in PPC globally.^43^, ^44^ Additionally, this method can inform the design and structure of future courses.^21^

Due to the ongoing COVID‐19 pandemic, this course was delivered virtually, including a novel bilingual asynchronous/synchronous format using an education platform (Cure4Kids). Several key features led to the success of this course. First, the curriculum was based on data from recent research identifying regional knowledge gaps^28^ with additional input from regional experts who understand the local context. In addition, expert regional bilingual facilitators allowed for contextually appropriate delivery of course content. Second, the unique bilingual asynchronous/synchronous format allowed participants to review course materials at their individual pace and simultaneous translation of live sessions allowed for real‐time discussion between English and Russian speakers. This ensured a better understanding of course content and encouraged the participation of all individuals, regardless of language skills, in the small‐group skill‐development activities. This bilingual format was successful in fostering a rich learning environment as demonstrated by participants highlighting the small group sessions and opportunities to learn from each other in their course evaluation. While other virtual training in palliative care have been developed,^45^, ^46^ our experience shows that this bilingual format is feasible and effective among diverse and geographically separate participants. The virtual format is also notably more cost‐effective, with decreased travel and lodging needs.

This work has several limitations. Only 75.9% of enrolled participants completed both pre‐ and postcourse assessments, preventing us from evaluating the knowledge acquisition of all participants. However, we wanted to accurately assess the impact of the entire course, and therefore, felt it appropriate to exclude those that did not complete all the course materials. Additionally, we do not evaluate improvements in clinical outcomes but rather self‐perceived improvement in clinician knowledge and confidence/comfort. Due to the limitation in capturing the clinical impact of improvement in knowledge, this is a common strategy employed in educational research.^47^, ^48^ Furthermore, the findings represented here relate to a single follow‐up timepoint. Additional monitoring and educational sessions will be needed to confirm the sustainability of identified improvements over time. Nevertheless, our unique training experience will be essential for building knowledge and expertise related to pediatric palliative care globally and to inform future educational initiatives.

The EPEC‐Pediatrics Eurasia course demonstrated that regional adaptation of the EPEC‐Pediatrics curriculum to a virtual asynchronous/synchronous platform delivered bilingually with local facilitators is feasible and effective. We plan to repeat this course annually with improvements from participant feedback. This successful experience can be leveraged to address learning gaps in PPC globally and overcome challenges arising from physical distance, language barriers, and the ongoing pandemic. Additionally, this feasible and effective educational strategy can inform the development of future curricula to improve provider knowledge globally.

## DISCLAIMER

This work including the preparation and facilitation of the educational conference as well as the preparation of the manuscript occurred prior to February 24, 2022.

## AUTHOR CONTRIBUTIONS

MJM, BE, TY, AA, and JNB developed the idea. MJM, BE, TY, AA, and JNB verified the data. MJM, BE, TY, HW, RR, and MD did the data analysis. MJM drafted the manuscript and prepared tables and figures. All the authors contributed to the interpretation of findings, editing of the article, and approved the final submitted version. All the authors had full access to all study data and accept responsibility for the decision to submit for publication.

## FUNDING INFORMATION

This project was supported by the American Lebanese Syrian Associated Charities (ALSAC).

## CONFLICT OF INTEREST

The authors declared that they have no conflict of interest.

## Supporting information


Appendix S1

## Data Availability

Data are available upon reasonable request from the authors.
