# Supplementary material for: Regional adaptation of the education in palliative and end‐of‐life Care Pediatrics (EPEC‐Pediatrics) curriculum in Eurasia
Source: Cancer Med. 2022 Sep 8;12(3):3657–69. doi: 10.1002/cam4.5213 (PMC9939085; doi:10.1002/cam4.5213)
Supplement: Supplementary file 1 — Appendix S1 [file CAM4-12-3657-s001.docx]

**Supplemental Tables and Figures:**

**Regional** **Adaptation of a Virtual Training in Pediatric Palliative Care: Education in Palliative and End-of-life Care-Pediatrics (EPEC-Pediatrics) in Eurasia**

Michael J. McNeil^1^, Bella Ehrlich^1,2^, Taisiya Yakimkova^1^, Huiqi Wang^1^, Volha Mishkova^3^, Karen Williams^1^, Zhanna Bezler^4^, Meenakshi Devidas^1^, Ella Kumirova^5,6,7,8,9^, Narine Movsisyan^10^, Arshia Madni^1^, Georgia Chatman^1^, Paola Nunez ^1^, Jason Sonnenfelt^1^, Baglan Baizakova^11^, Marina Borisevich^3^, Ximena Garcia Quintero^1^, Indira Erimbetova^12^, Rodica Golban^13^, Brandi Kirby^1^, Nadezhda Sakhar^14^, Alisa Volkova^15^, Radhikesh Ranadive^1^, Daniel Moreira^1^ Stefan J. Friedrichsdorf^16^, Joanne Wolfe^17^, Stacy Remke^18^, Joshua Hauser^19^, Justin N Baker^1^*, Asya Agulnik^1^*

**Table of Contents**

| **Item** | **Page** |
| --- | --- |
| **Supplemental Table 1:** High Yield EPEC-Pediatrics Modules for Eurasian Region | 2 |
| **Supplemental Table 2:** 5-day Workshop Schedule | 2 |
| **Supplemental Table 3:** Course Conceptual Framework | 3 |
| **Supplemental Figure 1:** Pre-Course Assessment | 4 |
| **Supplemental Figure 2:** Post-Course Assessment | 9 |
| **Supplemental Table 4:** Demographics of Participants Who Did Not Complete Course | 13 |
| **Supplemental Table 5:** Themes and Quotes | 14 |
| **Supplemental Table 6:** Individual Experience and Self-Assessment | 15 |

**Supplemental Table 1: High Yield EPEC-Pediatrics Modules for Eurasian Region**

| **Module 1** | What is Pediatric Palliative Care and Why Does it Matter? |
| --- | --- |
| **Module 2** | Child Development |
| **Module 5** | Self-Care for Professionals |
| **Module 7** | Communication and Planning |
| **Module 10** | Multi-Modal Analgesia |
| **Module 11** | Opioid Selection and Opioid Rotation |
| **Module 12** | Management of Neuropathic Pain Management and Adjuvant Analgesia |
| **Module 13** | Procedural Pain Management Strategies |
| **Module 14** | Chronic Complex Pain |
| **Module 16** | Management of Respiratory Symptoms |

**Supplemental Table 2: 5-day Workshop Schedule**

| **Time** | **Monday** | **Tuesday** | **Wednesday** | **Thursday** | **Friday** |
| --- | --- | --- | --- | --- | --- |
| **7:00-7:55 CST**  **15:00-15:55 MSK** | What is Pediatric Palliative Care and Why Does it Matter? | Multimodal Analgesia | Opioid Selection and Rotation | Neuropathic Pain Management and Adjuvant Analgesia | Procedural Pain |
| **7:55-9:00 CST**  **15:55-17:00 MSK** | Child Development or Respiratory Symptoms Small Group | Child Development or Respiratory Symptoms Small Group | Question and Answer Session/ Teaching How to Teach | Self-Care or Communication Small Group | Self-Care or Communication Small Group |

**Supplemental Table 3 Course Conceptual Framework**

| **Course Concept** | **Pre-Course Assessment Statement** | **Post-Course Assessment Statement** |
| --- | --- | --- |
| **Knowledge Acquisition According to WHO Guidance** | Early integration of palliative care for all children diagnosed with cancer would decrease patient suffering. | Early integration of palliative care for all children diagnosed with cancer would decrease patient suffering. |
| **Standard Accepted Components of Palliative Care** | The role of palliative care in the care of children with cancer is (please choose all that apply): | The role of palliative care in the care of children with cancer is (please choose all that apply): |
| **Ideal versus Actual Timing of Palliative Care** | Assuming unlimited resources, when do you think is the **ideal t**iming of **initial** palliative care consultation for a child with cancer (please choose all that apply): | Assuming unlimited resources, when do you think is the **ideal t**iming of **initial** palliative care consultation for a child with cancer (please choose all that apply): |
| **Comfort and Confidence in Providing Palliative care** | I feel confident providing **grief and bereavement care** to the families of children who die. | I feel confident providing **grief and bereavement care** to the families of children who die. |

**Supplemental Figure 1: Pre-Course Survey**

**Demographics**

1. Please indicate your full name. (First, Last)

2. Please indicate your email address.

3. What is the country in which you currently practice medicine?

Azerbaijan

Armenia

Belarus

Georgia

Kazakhstan

Kyrgyzstan

Moldova

Mongolia

Poland

Romania

Russia

Serbia

Tajikistan

Turkmenistan

Uzbekistan

Ukraine

Other: (fill in the blank)

4. What is your age?

<35

35 to 50

51 to 65

>65

5. What is your sex?

Female

Male

6. Please indicate your primary medical specialty:

Pediatric hematology and/or oncology

Pediatric anesthesia/ intensive care

Pediatric surgery

Pediatric palliative care

General pediatrician

Adult hematology and/or oncology

Adult anesthesia/ intensive care

Adult surgery

Adult palliative care

General physician

Other (describe)

7. Which kind of primary institution do you work in?

General Hospital

Children’s Hospital

Cancer Hospital

Children’s Hospice

Other (describe)

8. How many years of experience in practice do you have since graduating medical school?

0-5 years

6-10 years

11-15 years

16-20 years

21+ years

9. Prior to this course, have you received any formal training in palliative care?

Yes

No

If Yes (check all that apply):

Undergraduate/ medical school course

Postgraduate course without official certification

Certificate course

Residency or fellowship in palliative care

Other: (Please describe below)

10. Do you have access to a pediatric palliative care expert for consultation in your practice? (Yes/No)

If yes, please check the type(s) of palliative care experts available:

Physician, nurse, social worker, psychologist

11. How many pediatric patients in your care (less than 18 years old) died in the last 12 months?

Categories (0, 1-5, 6-10, 11-20, 21+)

**Perspectives**

*Please rate the extent to which you agree with the following statements about* ***pediatric oncology patients*** *receiving* ***palliative care****. Please check one box per line:*

|  | ***Strongly Disagree*** | ***Somewhat Disagree*** | ***Neutral*** | ***Somewhat***  ***Agree*** | ***Strongly***  ***Agree*** |
| --- | --- | --- | --- | --- | --- |

12. It is difficult to know when a patient with cancer would most benefit from meeting the palliative care team.

13. Children with advanced and incurable cancer often suffer at the end-of-life.

14. Early consultation with palliative care causes increased parental burden and anxiety.

15. Palliative care can be integrated with disease-directed therapy.

16. Involving palliative care suggests the oncologist has failed in the mission to cure the patient.

17. Palliative care for children with cancer can be delivered by health care workers of all disciplines, not only by palliative care specialists.

18. Palliative care is synonymous with “end-of-life” care.

19. Involvement of palliative care during cancer therapy gives greater attention to quality of life and symptom management (e.g. pain, constipation, dyspnea, fatigue).

20. Involvement of palliative care undermines the role of the pediatric oncologist as the physician in charge of patient care.

21. Children with cancer who receive palliative care die earlier than those who do not.

22. Early integration of palliative care for all children diagnosed with cancer would decrease patient suffering.

23. Early integration of pediatric palliative care with cancer care would improve interdisciplinary communication.

24. Palliative care is incompatible with curative care.

25. Involving the palliative care team early has negative effects on the relationship between the oncologist and the patient and family.

26. Palliative care is appropriate at any stage of treatment in a child with high-risk cancer.

27. Administering opioids to patients in pain hastens death due to respiratory depression.

28. There are situations where it is in a dying child’s best interest to remove mechanical ventilation if in alignment with the family’s wishes.

**Individual Experiences:**

***Please rate the frequency of the following statements about your individual experiences with pediatric oncology patients. Please check one box per line:***

|  | ***Never*** | ***Rarely*** | ***Sometimes*** | ***Often*** | ***Always*** |
| --- | --- | --- | --- | --- | --- |

29. In my setting, I have felt that involvement of palliative care has occurred too late in the treatment of a child with cancer.

30. In my setting, I have acted against my conscience by providing **aggressive cancer-directed treatment** to a pediatric oncology patient with advancing disease.

31. I have felt burdened by my inability to control the suffering of children at the end-of-life.

32. I feel burned out by my work.

33. I have become more callous toward people since I took this job.

***Please rate the extent to which you agree with the following statements about your individual experiences with pediatric oncology patients. Please check one box per line:***

|  | ***Strongly Disagree*** | ***Somewhat Disagree*** | ***Neutral*** | ***Somewhat***  ***Agree*** | ***Strongly***  ***Agree*** |
| --- | --- | --- | --- | --- | --- |

34. I feel confident assessing and treating the **physical needs** of pediatric patients with serious incurable illness.

35. I feel confident assessing and treating the **emotional needs** of pediatric patients with serious incurable illness and their families.

36. I feel confident providing **grief and bereavement care** to the families of children who die.

**Multiple choice:**

*Please choose* ***all*** *that apply for every option.*

37. The role of palliative care in the care of children with cancer is (please choose all that apply):

To aid in reducing pain and suffering related to disease and/or treatment

- To provide psychological support to the patient and their family
- To provide spiritual support to the patient and their family
- To aid in family decision-making around treatment options
- To help clarify the goals of care of the patient and family
- To help communicate bad news to patients and families
- To aid in communication between the patient, family, and medical teams
- To assist with transitions from the hospital to hospice or home at end-of-life
- Palliative care has no role in the care of children with cancer
- Other: Please describe

38. When does **initial** palliative care consultation for a child with cancer **typically** occur in your setting (please choose all that apply):

- At the time of cancer diagnosis for all patients
- At the time of cancer diagnosis for patients at high-risk of relapse or progression
- At the time of disease relapse or progression
- At the time of complex or high symptom burden (pain, suffering)
- When there are no longer curative therapeutic options available
- At the end of life
- Palliative care is typically not consulted for children with cancer (because it is not necessary or not available)

39. Assuming unlimited resources, when do you think is the **ideal t**iming of **initial** palliative care consultation for a child with cancer (please choose all that apply):

- At the time of cancer diagnosis for all patients
- At the time of cancer diagnosis for patients at high-risk of relapse or progression
- At the time of disease relapse or progression
- At the time of complex or high symptom burden (pain, suffering)
- When there are no longer curative therapeutic options available
- At the end of life
- Palliative care consultation is never necessary in pediatric cancer care

_________________________

Thank you for taking the time to complete the survey.

**Supplemental Figure 2: Post-Course Survey**

Please indicate your full name (First, Last)

Please indicate your email address.

**Perspectives**

*Please rate the extent to which you agree with the following statements about* ***pediatric oncology patients*** *receiving* ***palliative care****. Please check one box per line:*

|  | ***Strongly Disagree*** | ***Somewhat Disagree*** | ***Neutral*** | ***Somewhat***  ***Agree*** | ***Strongly***  ***Agree*** |
| --- | --- | --- | --- | --- | --- |

1. It is difficult to know when a patient with cancer would most benefit from meeting the palliative care team.

2. Children with advanced and incurable cancer often suffer at the end-of-life.

3. Early consultation with palliative care causes increased parental burden and anxiety.

4. Palliative care can be integrated with disease-directed therapy.

5. Involving palliative care suggests the oncologist has failed in the mission to cure the patient.

6. Palliative care for children with cancer can be delivered by health care workers of all disciplines, not only by palliative care specialists.

7. Palliative care is synonymous with “end-of-life” care.

8. Involvement of palliative care during cancer therapy gives greater attention to quality of life and symptom management (e.g. pain, constipation, dyspnea, fatigue).

9. Involvement of palliative care undermines the role of the pediatric oncologist as the physician in charge of patient care.

10. Children with cancer who receive palliative care die earlier than those who do not.

11. Early integration of palliative care for all children diagnosed with cancer would decrease patient suffering.

12. Early integration of pediatric palliative care with cancer care would improve interdisciplinary communication.

13. Palliative care is incompatible with curative care.

14. Involving the palliative care team early has negative effects on the relationship between the oncologist and the patient and family.

15. Palliative care is appropriate at any stage of treatment in a child with high-risk cancer.

16. Administering opioids to patients in pain hastens death due to respiratory depression.

17. There are situations where it is in a dying child’s best interest to remove mechanical ventilation if in alignment with the family’s wishes.

**Multiple choice:**

*Please choose* ***all*** *that apply for every option.*

18. The role of palliative care in the care of children with cancer is (please choose all that apply):

- To aid in reducing pain and suffering related to disease and/or treatment
- To provide psychological support to the patient and their family
- To provide spiritual support to the patient and their family
- To aid in family decision-making around treatment options
- To help clarify the goals of care of the patient and family
- To help communicate bad news to patients and families
- To aid in communication between the patient, family, and medical teams
- To assist with transitions from the hospital to hospice or home at end-of-life
- Palliative care has no role in the care of children with cancer
- Other: Please describe

19. When does **initial** palliative care consultation for a child with cancer **typically** occur in your setting (please choose all that apply):

- At the time of cancer diagnosis for all patients
- At the time of cancer diagnosis for patients at high-risk of relapse or progression
- At the time of disease relapse or progression
- At the time of complex or high symptom burden (pain, suffering)
- When there are no longer curative therapeutic options available
- At the end of life
- Palliative care is typically not consulted for children with cancer (because it is not necessary or not available)

20. Assuming unlimited resources, when do you think is the **ideal t**iming of **initial** palliative care consultation for a child with cancer (please choose all that apply):

- At the time of cancer diagnosis for all patients
- At the time of cancer diagnosis for patients at high-risk of relapse or progression
- At the time of disease relapse or progression
- At the time of complex or high symptom burden (pain, suffering)
- When there are no longer curative therapeutic options available
- At the end of life
- Palliative care consultation is never necessary in pediatric cancer care

**Self-Assessment**

***Please rate the extent to which you agree with the following statements about your individual experiences. Please check one box per line:***

|  | ***Strongly Disagree*** | ***Somewhat Disagree*** | ***Neutral*** | ***Somewhat***  ***Agree*** | ***Strongly***  ***Agree*** |
| --- | --- | --- | --- | --- | --- |

21. I feel confident assessing and treating the **physical needs** of pediatric patients with serious incurable illness.

22. I feel confident assessing and treating the **emotional needs** of pediatric patients with serious incurable illness and their families.

23. I feel confident providing **grief and bereavement care** to the families of children who die.

**As a result of my participation in this educational seminar…**

24. I feel more confident in my knowledge of opioid prescription and pain management for children with cancer.

25. I feel more confident in my ability to control the suffering of children at the end-of-life.

26. I better understand possible solutions on how to provide better palliative care for children with cancer.

27. I better understand how to hold difficult conversations with patients and their families and participate in decision-making and planning for the end of life.

**EPEC-P Course assessment**

***Please rate the extent to which you agree with the following statements about the EPEC-P course. Please check one box per line:***

|  | ***Strongly Disagree*** | ***Somewhat Disagree*** | ***Neutral*** | ***Somewhat***  ***Agree*** | ***Strongly***  ***Agree*** |
| --- | --- | --- | --- | --- | --- |

28. The EPEC-Pediatrics course provided me with new knowledge and skills that are relevant for my clinical practice.

29. The EPEC-Pediatrics course provided me with knowledge and skills that will CHANGE my clinical practice.

30. Please provide example(s) of something that you will do differently in the future (if applicable). [Open response]

31. Please provide example(s) of new concepts that were previously unclear or unknown (if applicable)

32. What was your favorite part of the EPEC-Pediatric course?

33. How would you improve the EPEC-Pediatrics course (e.g. structure, Zoom organization, content)?

34. In terms of your expectations, the EPEC-Pediatrics course was:

-far below

-moderately below

- slightly below

-met expectations

-slightly above

-moderately above

-far above

**Supplemental Table 4: Demographics of Participants Who Did Not Complete Course**

| **Demographics Overall Sample**  **(n=11), No. (%)**  **Country** | |
| --- | --- |
| Kazakhstan | 2 (18.2) |
| Romania | 3 (27.3) |
| Russia | 2 (18.2) |
| Ukraine | 3 (27.3) |
| Uzbekistan | 1 (9.1) |
| **Age** | |
| <35 | 5 (45.4) |
| 35 to 50 | 4 (36.4) |
| 51 to 65 | 2 (18.2) |
| >65 | 0 |
| **Sex** | |
| Female | 10 (90.9) |
| Male | 1 (9.1) |
| **Primary medical specialty** | |
| Pediatric hematology and/or oncology | 5 (45.4) |
| Pediatric anesthesia/ intensive care | 1 (9.1) |
| General pediatrician | 4 (36.4) |
| Adult anesthesia/ intensive care | 1 (9.1) |
| **Primary institution** | |
| Children's Hospital | 5 (45.4) |
| Cancer Hospital | 3 (27.3) |
| General Hospital | 2 (18.2) |
| *Other: (please describe) | 1 (9.1) |
| **Years of experience** | |
| - 1. ears | 6 (54.6) |
| >11 years | 5 (45.4) |
| **Previous training in palliative care** | |
| No | 10 (90.9) |
| Yes | 1 (9.1) |
| **Access to palliative care consultation** | |
| No | 9 (81.8) |
| Yes | 2 (18.2) |
| **How many pediatric patients in your care (less than 18 years old) died in the last 12 months?** | |
| 0 patients | 4 (36.4) |
| 1-5 patients | 5 (45.4) |
| >6 patients | 2 (18.2) |

*National Research Center

**Supplemental Table 5: Themes and Quotes**

| **Pain and Symptom Management** | **“**I will try to think more about comfort of the patient and improve reduce the pain in patient. Sometimes we think, that the treatment and the curable therapy is the most important... I use opioids, so I haven't any resistance to give them. But it was interesting, that small doses of naloxone reduce the side effects of morphine, so I will use it in my practice.” |
| --- | --- |
| **Psychosocial Support** | **“**More carefully care for psychological nuances in the period of life of the child during my work, communication with the child.” |
| **Communication Skills** | “I have changed my approach to how I can communicate to patients and their families that their disease is incurable. I recognized in which situations I was unable to recognize the importance of the child's consent to treatment and procedures. I understood the importance and possible ways of recognizing the impact of developmental status on the experience of childhood illness and identifying family preferences for involving children in decision-making.” |
| **Earlier Integration of Palliative Care** | For me, the statement that the involvement of a palliative team is possible at the time of diagnosis was a discovery. I agree that talking with parents about palliative care early will facilitate communication at different stages of the disease. I am going to inform doctors about this, to spread the necessary information about palliative for parents. It's not easy, because the mentality and attitude towards the very word "palliative" is not very simple in our country, and the "doctor-patient" communication is very upsetting” |
| **Self-Care** | “I have been working in oncology for 28 years. I keep in touch with many already cured patients, as well as with the parents of children who have died. I value them, but I understand that I need to learn to love myself and devote more time to my family.” |
| **Small-Group Sessions** | “I liked the possibility of hearing all the voices, with less or more experience, and the fact that it was no bad answer. All the doctors/lecturers were amazing, you can tell from the first minute that they can do anything they want in life because they are smart, confident, hard-working and love their work!” |

**Supplemental Table 6: Individual Experience and Self-Assessment:**

|  | *Pre*  *(Individual Experiences) (N=44)* | *Post*  *(Self-Assessment) (N=44)* | *P-value^a^* |
| --- | --- | --- | --- |
| **I feel confident assessing and treating the physical needs of pediatric patients with serious incurable illness.** | | | |
| Disagree | 6 (13.6) | 9 (20.5) | 0.0336 |
| Neutral | 12 (27.3) | 2 (4.5) |  |
| Agree | 26 (59.1) | 33 (75.0) |  |
| **I feel confident assessing and treating the emotional needs of pediatric patients with serious incurable illness and their families.** | | | |
| Disagree | 7 (15.9) | 4 (9.1) | 0.2276 |
| Neutral | 5 (11.4) | 2 (4.5) |  |
| Agree | 32 (72.7) | 38 (86.4) |  |
| **I feel confident providing grief and bereavement care to the families of children who die.** | | | |
| Disagree | 10 (22.7) | 6 (13.6) | 0.6649 |
| Neutral | 15 (34.1) | 8 (18.2) |  |
| Agree | 19 (43.2) | 30 (68.2) |  |

^a^: Symmetry Test
